# A cross-sectional study investigating lifestyle and weight perception of undergraduate students in southern Italy

**DOI:** 10.1186/s12889-019-7695-z

**Published:** 2019-10-21

**Authors:** Francesca Gallè, Elita Anna Sabella, Giovanna Da Molin, Giorgio Liguori, Maria Teresa Montagna, Giovanni Battista Orsi, Giuliana Valerio, Christian Napoli

**Affiliations:** 1 Department of Movement Sciences and Wellbeing, University of Naples “Parthenope”, Via Medina n. 40, 80133 Naples, Italy; 2 Inter-University Research Centre “Population, Environment and Health”, University of Bari Aldo Moro, Piazza Umberto I, 1, 70121 Bari, Italy; 3 Department of Biomedical Science and Human Oncology, University of Bari Aldo Moro, Piazza G. Cesare 11, 70124 Bari, Italy; 4 Department of Public Health and Infectious Diseases, “Sapienza” University of Rome, Piazzale Aldo Moro 5, 00185 Rome, Italy; 5 Department of Medical Surgical Sciences and Translational Medicine, “Sapienza” University of Rome, Via di Grottarossa 1035/1039, 00189 Rome, Italy

**Keywords:** Lifestyle, Undergraduate students, Smoking, Alcohol consumption, Physical activity, Nutritional status

## Abstract

**Background:**

The aim of the study was to explore lifestyle of undergraduate students in southern Italy and to investigate their determinants.

**Methods:**

An anonymous, web-based questionnaire investigating weight and its perception, smoking and alcohol use, achievement of recommended levels of physical activity and time spent daily in screen-based sedentary behaviors was administered to students attending two universities in southern Italy. Age, gender, city, type of degree course attended, occupational status and residential status were considered as possible demographic determinants.

**Results:**

The majority of the participants reported a normal weight (71.2%), were non-smokers (66.6%), occasional alcohol consumers (60.5%) and insufficiently active (62.6%), with a reported mean screen time exceeding 2 h per day. Gender, city, type of degree course and occupational status were found to be associated with lifestyle by regression analysis. Normal weight, overweight and obese students were generally properly aware of their weight condition; however, weight misperceptions were registered among normal and underweight undergraduates.

**Conclusions:**

This study highlighted some critical issues regarding lifestyles of university students that suggest the need for health promotion interventions targeted mainly on physical activity.

## Background

In the last decades, non-communicable chronic diseases such as obesity, cardiovascular diseases, cancer and type 2 diabetes became the most important causes of death globally. The role of lifestyle in the development of these diseases has been widely recognized; in particular, inadequate diet, inactivity, smoke and alcohol consumption represent the main behavioral risk factors [1].

In Italy, the epidemiological systems activated to monitor inhabitants’ health conditions and habits (“OKkio alla salute” for children, www.epicentro.iss.it/okkioallasalute/; “Health Behaviour in School-aged Children” for adolescents, http://www.hbsc.unito.it/it/; “Passi” for adults, http://www.epicentro.iss.it/passi/en/english.asp) show that both unhealthy behaviors (smoke, alcohol consumption, inactivity) and related health conditions and diseases (overweight/obesity, cardiovascular diseases, type 2 diabetes) are more common in Sothern regions. In addition, it is reported a low adherence of the Italian population to the Mediterranean dietary pattern, which is known to decrease morbidity and mortality related to chronic diseases [2–5]. The low adherence to the Mediterranean diet in the Italian population is also associated to overweight/obesity or to other unhealthy habits, such as insufficient levels of physical activity (PA).

In young people, the transition from adolescence to adulthood corresponds to the achievement of independence and this may lead to the development or the consolidation of unhealthy habits [6]. University students, in particular, seem to be exposed to unhealthy sedentary and dietary habits, especially if living away from home [7–9]: a previous survey carried out in northern Italy highlighted some clear differences in food consumption, sport practice and body weight perception between undergraduates living with families and those living alone [10]. Due to the typical sedentary lifestyle of the students and their difficulties in finding free time, the amount of PA they reach during the week is frequently lower than the World Health Organization (WHO) recommendations [11–13]. In addition, they spent also a lot of time in screen-based sedentary behaviors such as watching television, playing videogames, studying/playing computer [14].

Some experiences reported also gender differences regarding undergraduates’ lifestyle and weight status, with females showing more frequently a normal weight and lower PA levels than males [13, 15].

Therefore, the promotion of healthy habits in this population group should be considered in a public health prospective. In Italy, many dietary guidelines have been produced, and several educational programs regarding nutrition, smoking and alcohol consumption are used to be implemented in the schools, while no similar interventions are targeted to university students.

In light of this scientific background, the aim of this study was to evaluate nutritional status and weight perception, together with health-related behaviors - tobacco smoking, alcohol use, PA level, and screen time - in a sample of undergraduate students attending two universities in southern Italy. In order to identify possible lifestyle determinants to take into consideration for future promotion interventions, the declared weight condition and behaviors were analyzed with regard to demographic variables.

## Methods

This cross-sectional study was carried out across the academic years 2015–2016 and 2016–2017 in the University of Naples Parthenope and in the University of Bari Aldo Moro by collecting undergraduates’ information through a web-based questionnaire.

The investigation was performed in accordance with the World Medical Association Declaration of Helsinki. It did not include any experiment on human or biological human samples, nor research on identifiable human data. The research was conducted anonymously (according to the Italian Legislative Decree no. 196/2003 concerning the protection of personal data).

Therefore, no identifiable personal data are reported. While accepting to respond to the questionnaire, students expressed their informed consent to participate to the investigation.

### Participants and settings

All the students attending the degree courses in the two universities were invited to take part to the investigation through an e-mail containing a brief description of the aim of the study and the guarantee about the anonymity of data collection and treatment, and a link to the web-based questionnaire created through the online survey tool SurveyMonkey.

The minimum sample size calculation was performed considering the estimated prevalence of smoke, alcohol use, insufficient PI and overweight/obesity in the Italian general population as reference. A sample of at least 217–375 individuals would be required to investigate the above mentioned variables in the students’ population examined, assuming a 5% alpha error and with 80% power.

### Questionnaire

In order to encourage the students’ participation, the web-based questionnaire was designed to be brief and user friendly. It included a first part regarding demographic characteristics such as gender, age, degree course, occupational (jobbing/employed or not) and living (residing in the area/not residing but living in the area/commuter) condition; students were also asked to self-report their height and weight values in order to calculate their Body Mass Index and related weight status (underweight/normal weight/overweight/obese) according to the WHO classification (http://www.euro.who.int/en/health-topics/disease-prevention/nutrition/a-healthy-lifestyle/body-mass-index-bmi), together with a personal judgement regarding their weight (“my weight is right”/I should gain weight/I should lose weight). The second part of the questionnaire was aimed to collect information regarding the lifestyles of participants: questions concerned tobacco (non-smoker/quitter/smoker) and alcohol use (never/occasionally/1–2 times per week/about every day), weekly PA level (practicing at least 150 min of sport/PA per week or not) and sedentary behavior (minutes of screen time per day).

### Statistical analysis

A descriptive analysis was carried out to highlight demographic characteristics of the whole sample and possible differences between the subgroups from the two cities. In addition to the classification resulting from the questionnaire structure, participants were also classified for age (18–21, 22–24, ≥ 25 years) and type of degree course (life science/others). This last classification was made assuming that life sciences students were more acquainted with healthy lifestyle recommendations, being that included in their curricula.

Continuous outcomes were reported as mean ± SD; data regarding conditions, behaviors and opinions were expressed as percentage values related to the answers proposed, calculated on the total of respondents.

The Student’s *t* test was used to compare mean age and BMI values between the two subgroups; the other variables were grouped in categories and compared through the chi-squared test. In order to explore possible differences between genders regarding BMI and weight perception, number and percentages of males/females belonging to the different BMI categories and those considering their weight normal, insufficient or excessive were compared through the chi-squared test.

Multinomial logistic regression analyses were performed to evaluate the possible association between demographic variables and lifestyle behaviors.

The outcome binary variables were built as follows:

- BMI, attributing the value 0 to the students who were underweight/normal weight and 1 to those who were overweight/obese
- Smoke, attributing the value 0 if the student was not a smoker, and the value 1 otherwise;
- Alcohol, considering the value 0 if the student did not consume alcohol and 1 otherwise;
- PA, with the value 0 if the student declared to reach at least 150 min per week and 1 otherwise;
- Screen time, with the value 0 attributed to the participants who declared a daily screen time ≤ 120 min and 1 to those who exceeded this value.

All the outcomes were investigated separately through a backward selection of the independent demographic variables (age class, gender, city, type of degree course, occupational status, residential status). Odds Ratios (ORs) and 95% Confidence Interval (CI) of differences between categories were considered.

A value of *p* < 0.05 was assumed as significance level. Data were analyzed with IBM SPSS version 23 for Windows (SPSS, Chicago, IL, USA).

## Results

The whole student population of both the University Parthenope and the University Aldo Moro (*n* = 59,779) were invited to participate to the study. A total of 1760 students (710 from Naples, 1050 from Bari; response rate 2.9%) fulfilled the questionnaire. A total of 448 students (25.5%) attended a degree course in the area of life science, while the remaining 1311 (74.5%) attended a degree course in other areas.

The general characteristics of the total sample and those of participants from the two samples are reported in Table 1. As shown, the two subgroups differed significantly for all the demographic variables considered, but the area of course degree: students attending life science degree courses represented about a quarter of both samples.

**Table 1:** General characteristics of the cohort pooled in the total sample and separated by origin with correspondent *p* values

	Total *(n = 1760)*	Naples *(n = 710)*	Bari *(n = 1050)*	*p* value
Mean age ± SD *years*	23.4 ± 4.09	24 ± 4.67	22.9 ± 3.58	< 0.001^a^
(range)	(18–60)	(18–60)	(19–57)	
Age class *n (%)*				
18–21	617 (35)	215 (30.3)	402 (38.3)	< 0.001^b^
22–24	661 (37.6)	255 (35.9)	406 (38.7)	
≥ 25	482 (27.4)	240 (33.8)	242 (23)	
Gender *n (%)*				
male	768 (43.6)	75 (52.8)	393 (37.4)	< 0.001^b^
female	992 (56.4)	335 (47.2)	657 (62.6)	
Degree course				
Life sciences	448 (25.5)	173 (24.4)	275 (26.2)	0.41^b^
Others	1311 (74.5)	537 (75.6)	774 (73.8)	
Occupational status				
jobbing/employed	663 (37.7)	343 (48.3)	320 (30.5)	< 0.001^b^
unemployed	1097 (62.3)	367 (51.7)	730 (69.5)	
Residential status				
residing in the area	411 (23.4)	204 (28.8)	207 (19.7)	< 0.001^b^
commuter	345 (19.6)	94 (13.2)	251 (23.9)	
not residing but living in the area	1004 (57)	412 (58)	592 (56.4)	
Smoke				
non-smoker	1173 (66.6)	469 (66.1)	704 (67)	0.90^b^
quitters	154 (8.8)	64 (9)	90 (8.6)	
smoker	433 (24.6)	177 (24.9)	256 (24.4)	
Alcohol use				
never	231 (13.1)	102 (14.4)	129 (12.3)	0.20^b^
occasionally	1064 (60.5)	437 (61.6)	627 (59.7)	
1–2 times/week	409 (23.2)	148 (20.8)	261 (24.9)	
vabout every day	56 (3.2)	23 (3.2)	33 (3.1)	
Physical activity				
inactive	1101 (62.6)	411 (57.9)	690 (65.7)	0.001^b^
active	659 (37.4)	299 (42.1)	360 (34.3)	
Mean BMI ± SD (kg/m^2^)	22.8 (3.4)	23.5 (3.4)	22.3 (3.4)	1.95^a^
BMI category	129 (7.4)	16 (2.3)	113 (10.8)	< 0.001^b^
underweight	1240 (71.2)	494 (71.5)	746 (71)	
normal weight	313 (18)	148 (21.4)	165 (15.7)	
overweight	59 (3.4)	33 (4.8)	26 (2.5)	
obese				
Mean daily screen time (min) ± SD	143 ± 140	168 ± 137.4	128 ± 139.5	< 0.001^b^

^a^ Student’s *t* test; ^b^ χ^2^ test

Mean BMI did not differ significantly between the two groups, while the analysis of weight category showed a significantly higher proportion of overweight/obese individuals among Neapolitan students.

As for lifestyle, the largest percentage of the participants declared to be non-smoker and occasional alcohol consumer; the majority of the total sample was also insufficiently active and reported screen time over 120 min per day. The same findings were shown in the two subgroups, however a slightly lower percentage of inactive students (57.9 vs 65.7%, *p* = 0.001) and a longer screen time (168 vs 129 min, *p* = < 0.001) were registered in Naples vs Bari.

Significant differences were found between genders regarding BMI classes and weight perception (Table 2): although higher percentages of overweight/obese subjects were found among males (29.9%), the highest number of participants who considered their weight excessive was registered among females (48.7%).

**Table 2:** Gender differences in BMI category and weight perception of participants with correspondent *p* values from chi square test

	Gender		*p* value
	Male *n = 768*	Female *n = 992*	
BMI category *n (%)*			
Underweight	14 (1.8)	115 (11.7)	< 0.001
Normal weight	520 (68.2)	720 (73.5)	
Overweight	187 (24.5)	126 (12.9)	
Obese	41 (5.4)	18 (1.8)	
Weight perception *n (%)*			
My weight is right	340 (44.3)	397 (40)	< 0.001
I should gain weight	122 (15.9)	112 (11.3)	
I should lose weight	306 (39.8)	483 (48.7)	

**Fig. 1:**
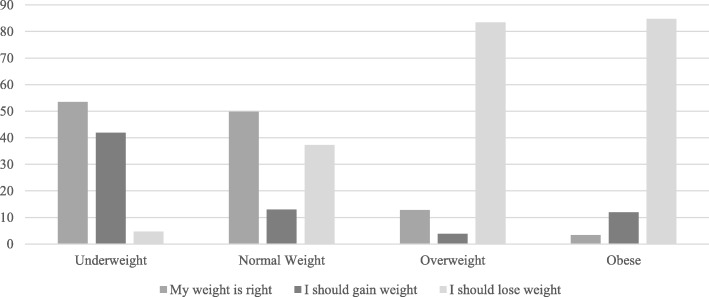
Judgements expressed by participants about their weight status grouped per BMI category of respondents

Figure 1 shows the participants’ opinions regarding their own weight, grouped for BMI category of respondents. It is possible to notice that the majority of the normal weight, overweight and obese students was properly aware of their own condition; however, there was a great part of normal-weight participants who considered their weight excessive, while the majority of underweight participants considered their nutritional status right.

The results of the logistic regression analyses performed on the 5 lifestyle variables are shown in Table 3. Age and residential status do not seem to influence unhealthy behaviors. On the contrary, attending life science courses and being employed seems to hinder smoking habit, while being male and coming from Bari may favor alcohol consumption.

**Table 3:** Results of logistic regression analyses carried out on the lifestyles assumed as dependent variables

	Dependent variables				
Independent variables	BMI	Smoke	Alcohol	PA	Screen time
	OR (CI 95%)				
Age class					
18–21	*Reference*	*Reference*	*Reference*	*Reference*	*Reference*
22–24	1.11 (0.83–1.49)	1.35 (1.04–1.76)	0.92 (0.73–1.15)	1.09 (0.86–1.37)	1.13 (0.90–1.43)
≥ 25	1.51 (1.11–2.06)	1.18 (0.88–1.59)	0.81 (0.63–1.05)	1.24 (0.95–1.61)	0.98 (0.75–1.28)
Gender					
female	*Reference*	*Reference*	*Reference*	*Reference*	*Reference*
male	2.29 (1.80–2.92)*	0.99 (0.80–1.25)	1.86 (1.50–2.32)*	0.66 (0.54–0.81)*	1.70 (1.40–2.09)*
City					
Bari	*Reference*	*Reference*	*Reference*	*Reference*	*Reference*
Naples	1.34 (1.04–1.70)*	0.95 (0.75–1.20)	0.68 (0.54–0.86)*	0.82 (0.66–1)	1.98 (1.61–2.44)*
Degree course					
others	*Reference*	*Reference*	*Reference*	*Reference*	*Reference*
life sciences	0.75 (0.57–0.99)*	0.55 (0.41–0.72)*	0.72 (0.56–0.93)	0.54 (0.43–0.67)*	0.87 (0.69–1.09)
Occupational status					
unemployed	*Reference*	*Reference*	*Reference*	*Reference*	*Reference*
jobbing/employed	0.85 (0.66–1.09)	0.66 (0.52–0.83)*	0.77 (0.62–0.97)	1.53 (1.24–1.89)*	1.19 (0.96–1.47)
Residential status					
residing in the area	*Reference*	*Reference*	*Reference*	*Reference*	*Reference*
commuter	1.16 (0.80–1.68)	1.05 (0.75–1.46)	0.82 (0.58–1.14)	1.38 (1.01–1.87)	1.10 (0.81–1.50)
not residing but living in the area	1.14 (0.85–1.52)	0.84 (0.64–1.10)	0.91 (0.70–1.19)	1.10 (0.86–1.39)	1.01 (0.79–1.29)

*p < 0.05

Male gender seems to be also a determinant for higher BMI and screen time, as well as living in Naples, but not for insufficient PA. Attending life science courses looks like a positive factor also for PA and BMI. The condition of student worker was associated with PA levels lower than those recommended.

## Discussion

The information collected in this study underline some critical issues regarding undergraduates’ lifestyle, especially if compared with data from the Italian general population. The percentage of non-smoker students was higher than that reported by the Italian Institute of Statistics for individuals aged 20–24 years in 2016 (66.6 vs 63.5%) [16]. Instead, the percentage of those who did not report any alcohol consumption in our investigation was lower than that reported for consumption between meals by the Italian population of the same age class (13.1 vs 26.8%) [16]. However, these values are consistent with the low percentage of smokers and with the high percentage of alcohol users registered among the Italian graduates [16], which suggests the association between these lifestyles and the educational level.

Regarding to the other lifestyles analyzed, although a normal weight was reported by the majority of the sample, it should be noted that the percentage of overweight/obese students was greater than that of the general population (21.4 vs 17.4%) and almost double than that recently reported by Teleman et al. in a sample of students from universities of Center and North Italy (11.2%) [15, 16]. In general, the largest proportion of the students reported a weight perception which matches to their declared weight condition, even if misperceptions were registered in part of normal and underweight students. Considering that females showed lower proportions of overweight/obese respect to males, and that they expressed an higher desire to lose weight, it is possible that most of these misperceptions were sustained by female students. This is in line with other investigations, which reported gender discrepancies in self-reported and desired weight [17–19].

More than 62% of participants were insufficiently active. This value is consistent with the levels of insufficient PA registered in the Italian population, which increases from 57 to 61.7 to 68.1% among individuals aged 18–19, 20–24 and 25–34 years respectively, confirming the reduction of PA/sport practice experienced since the beginning of the adult age and highlighting the necessity of promoting PA in this period of life [16]. In addition, in our study undergraduates reported more than 2 h per day spent in screen-based sedentary activities. Insufficient PA levels and high levels of inactivity among undergraduates, even in coexistence with normal weight, are in line with other surveys [12–14]. The regression analysis showed that male gender was associated to unhealthy nutritional status and inactive lifestyle, while attending the Neapolitan university seems to be associated with higher BMI and screen time but not with insufficient PA. However, it should be considered that being employed resulted positively associated to low levels of PA, which suggests that working students encounter greater difficulties in finding free time for recreational or structured activities to meet WHO recommendations: the reason why Neapolitan students, who were largely workers, showed good levels of PA is probably related with the fact that a great amount of them studied and were employed in the field of PA and sport (data not reported) [20, 21].

The regression analysis confirmed also the gender differences regarding BMI and PA levels reported in previous studies [13, 15], while no significant associations were found between age and lifestyles, probably due to the narrow age range considered.

In 2015, Lupi et al. published the results of a survey aimed to assess dietary habits, sport practice and body weight perception in a sample of undergraduates attending medical and scientific courses at a university in northern Italy [10]. Their findings testified the difficulties that students, especially those living away from home, encounter in adopting healthy lifestyles. In our investigation, the condition of student living alone in the city of the university did not result determinant for unhealthy behaviors adoption, and belonging to life science degree courses appeared protective towards unhealthy behaviors. However, it should be considered that our sample was wider, had a different geographical origin and included also students attending other degree courses than those regarding life sciences. It is possible that these differences played a role in determining these different findings.

This study has some limitations. First of all, in order to increase the number of participants, we chose to submit a questionnaire short and quick to fill in; however, this implied the exclusion of some questions regarding, for example, the socio-economic status or the parents’ educational level.

In addition, information regarding weight and height, so as weekly PA levels, were referred by participants and not objectively measured by the investigators. Therefore, it is possible that self-reported measurements were not always accurate and this might have affected the comparison between perceived and actual weight.

At last, the study may have been affected by selection biases. It was aimed at exploring lifestyles of the university students, who represent a specific population group and are not representative of the whole population of young adults in southern Italy. Moreover, the response rate was very low; this is probably due to the scarce students’ compliance to accept an online invitation. However, it should be noted that the minimum sample size needed to investigate the selected variables was achieved and the proportions of students from life science and other degree courses reflected the one of the whole undergraduate populations of the two enrolled Universities (about 25 and 75% respectively).

Further investigations based on different collecting methods and including people not only attending Universities are needed to complete the lifestyle picture of young adults in this geographical area.

## Conclusions

This study represents a contribute to fill the gap in characterizing the lifestyle of undergraduates in southern Italy. The main findings highlight the need of health promotion interventions targeted to this specific population group and focused on specific issues. Interventions aimed at improving undergraduates’ knowledge towards unhealthy lifestyle consequences and motivational programs enhancing the adoption of healthy behaviors should be implemented. In particular, the low levels of PA underline that interventions focused on PA promotion and the reduction of inactivity may be useful for the studied population group as well as for the Italian general young adult population to reduce overweight/obesity and maintain health.

The weight misperception registered in the study requires further specific investigations.

## Data Availability

The datasets generated during the current study are available from the corresponding author on reasonable request.

## Abbreviations

BMI: Body Mass Index
PA: Physical Activity
WHO: World Health Organization
